# Disparities in Health Care Spending and Utilization Among Black and White Medicaid Enrollees

**DOI:** 10.1001/jamahealthforum.2022.1398

**Published:** 2022-06-10

**Authors:** Jacob Wallace, Anthony Lollo, Kate A. Duchowny, Matthew Lavallee, Chima D. Ndumele

**Affiliations:** 1 Yale School of Public Health, New Haven, Connecticut; 2 University of California, San Francisco; 3 Yale University, New Haven, Connecticut

## Abstract

**Importance:**

Administrative records indicate that more than half of the 80 million Medicaid enrollees identify as belonging to a racial and ethnic minority group. Despite this, disparities within the Medicaid program remain understudied. For example, we know of no studies examining racial differences in Medicaid spending, a potential measure of how equitably state resources are allocated.

**Objectives:**

To examine whether and to what extent there are differences in health care spending and utilization between Black and White enrollees in Medicaid.

**Design, Setting, and Participants:**

This cross-sectional study used calendar year 2016 administrative data from 3 state Medicaid programs and included 1 966 689 Black and White Medicaid enrollees. Analyses were performed between January 28, 2021, and October 18, 2021.

**Exposures:**

Self-reported race.

**Main Outcomes and Measures:**

Rates and racial differences in health care spending and utilization (including Healthcare Effectiveness Data and Information Set [HEDIS] access measures).

**Results:**

Of 1 966 689 Medicaid adults and children (mean [SD] age, 20.3 [17.1] years; 1 119 136 [56.9%] female), 867 183 (44.1%) self-identified as non-Hispanic Black and 1 099 506 (55.9%) self-identified as non-Hispanic White. Results were adjusted for age, sex, Medicaid eligibility category, zip code, health status, and usual source of care. On average, annual spending on Black adult (19 years or older) Medicaid enrollees was $317 (95% CI, $259-$375) lower than White enrollees, a 6% difference. Among children (18 years or younger), annual spending on Black enrollees was $256 (14%) lower (95% CI, $222-$290). Adult Black enrollees also had 19.3 (95% CI, 16.78-21.84), or 4%, fewer primary care encounters per 100 enrollees per year compared with White enrollees. Among children, the differences in primary care utilization were larger: Black enrollees had 90.1 (95% CI, 88.2-91.8) fewer primary care encounters per 100 enrollees per year compared with White enrollees, a 23% difference. Black enrollees had lower utilization of most other services, including high-value prescription drugs, but higher emergency department use and rates of HEDIS preventive screenings.

**Conclusions and Relevance:**

In this cross-sectional study of US Medicaid enrollees in 3 states, Black enrollees generated lower spending and used fewer services, including primary care and recommended care for acute and chronic conditions, but had substantially higher emergency department use. While Black enrollees had higher rates of HEDIS preventive screenings, ensuring equitable access to all services in Medicaid must remain a national priority.

## Key Points

**Question:**

Are there racial differences in health care spending and utilization for low-income individuals in the US who are covered by the Medicaid program?

**Findings:**

In this cross-sectional study of 1 966 689 Black and White Medicaid enrollees in 3 states, Black enrollees used fewer services, including primary care, and generated lower spending than White enrollees, but were more likely to utilize the emergency department for avoidable reasons. Differences persisted among enrollees residing in the same zip codes who were treated by the same health care professionals.

**Meaning:**

The results of this study suggest that stark differences in spending and primary care use exist between Black and White Medicaid enrollees, and additional steps to ensure equity are needed.

## Teaser

This cross-sectional study examines whether and to what extent there are differences in health care spending and utilization between Black and White enrollees in Medicaid.

## Introduction

Racial disparities in health care access and health are well documented in the US.^1,2,3,4,5,6,7,8^ While expanding health insurance coverage reduces disparities,^9,10,11,12,13,14,15^ substantial unexplained variation remains. Prior studies found that racial disparities persist among US children and adults with the same sources of health insurance^16,17,18,19,20^ and those treated by the same health systems.^21^

Administrative records indicate that more than half of Medicaid enrollees identify as belonging to a racial and ethnic minority group. However, despite the overrepresentation of underserved populations in Medicaid (and the national focus on health equity), disparities in access to and utilization of care within Medicaid remain understudied.^22^ Medicaid, as the primary source of coverage for historically underserved groups, could play a critical role in reducing racial disparities in care.^23^ Because Medicaid services are provided at no (or low) cost to enrollees, differential access based on ability to pay, which is often associated with race in the US,^24^ is less of a concern. However, racial disparities still arise in Medicaid because of the barriers erected by pervasive interpersonal discrimination and structural racism.^25,26,27,28,29^

Although studies have examined disparities in Medicaid for specific populations or conditions, to our knowledge, few have documented racial disparities using data from multiple states.^30,31,32^ We know of no studies that examine racial disparities in Medicaid spending, a measure of how equitably state resources are allocated. Moreover, the rise of Medicaid managed care (MMC), through which states contract with private plans to administer Medicaid benefits, has led to concerns that the incentives of plans to lower costs or avoid enrollees with more severe illness may exacerbate disparities, although recent evidence is limited and mixed.^33,34,35,36^

In this study, we assessed racial differences in health care spending and utilization for adults and children enrolled in Medicaid using administrative data collected from 3 states using MMC. We examined differences between racial and ethnic minority groups with and without adjusting for enrollee and area-level characteristics, such as age, sex, eligibility category, health status, and zip code.

## Methods

### Study Design and Population

We obtained enrollee-level administrative Medicaid data directly from 3 Southern or Midwestern states that operated MMC programs for the calendar year 2016 (the most recent year of data made available to us). Pursuant to agreements with state partners designed to protect the confidentiality of managed care plans and avoid out-of-context state comparisons, we do not have permission to identify the specific study states. States that were chosen were those with high data quality, racial diversity, and high MMC penetration.

Comparisons of the characteristics of study states with national averages indicated that the study states were broadly representative in terms of urbanicity and health insurance coverage patterns, but these states had a higher share of their populations with income levels at or below the poverty line, had a higher share of non-Hispanic Black residents, and were more reliant on MMC (eTable 1 in the Supplement). We restricted the sample to non-Hispanic Black (hereafter *Black*) and non-Hispanic White (hereafter *White*) enrollees because of data limitations in the coding of race and ethnicity and the smaller sample sizes of Asian, Hispanic, and American Indian/Alaska Native enrollees in the study states. This led to the exclusion of 23 175 Asian enrollees, 84 645 Hispanic enrollees, and 16 259 American Indian/Alaska Native enrollees. We also restricted the sample to non–dual-eligible enrollees and individuals continuously enrolled (ie, we removed enrollees with partial year enrollment) in Medicaid in 2016 (eFigure 1 in the Supplement). We stratified the primary analyses by children (age 0-18 years) and adults (19 years or older) because of distinct patterns of care and Medicaid eligibility pathways for these groups.

The Yale University institutional review board reviewed the study and deemed it exempt because we used retrospective deidentified data; informed consent was waived according to the Regulations for the Protection of Human Subjects. The study followed the Strengthening the Reporting of Observational Studies in Epidemiology (STROBE) reporting guidelines.

### Study Variables

From monthly Medicaid eligibility data, we obtained enrollees’ self-identified race (Black or White), age, sex, Medicaid eligibility category, and zip code (eMethods and eTable 2 in the Supplement). Because the extent of missing data on race varied by state, we performed sensitivity analyses stratified by state.

From administrative claims data, we constructed annual spending per enrollee (this was the sum of all payments to health care professionals, hospitals, and clinics) for all services and spending separately for prescription drugs and medical care. For utilization measures, we stratified by categories of service (eg, inpatient, primary care, and emergency department). We also constructed several measures of utilization of recommended services or other proxies for access, including the utilization of high-value therapeutic drug classes^37,38^ by enrollees with qualifying diagnoses (eMethods in the Supplement) and rates of avoidable (ie, nonemergency) emergency department use.^39^ In addition, we followed the Healthcare Effectiveness Data and Information Set (HEDIS), which is commonly used to evaluate performance in Medicaid, to construct measures of the receipt of recommended services for preventive care and acute and chronic conditions (eTable 3 in the Supplement). Measures were selected from the Medicaid Child and Adult Core Sets after we assessed whether they could be reliably derived from administrative claims.^40^

Using enrollee diagnoses in 2016, we created 141 indicators of enrollee risk based on the Health and Human Services Hierarchical Condition Category model, a concurrent risk adjustment model that uses diagnosis codes to categorize enrollees into clinically meaningful condition categories. We also attributed each enrollee to a usual source of care, which was the health care professional or medical institution with whom they had the most claims during 2016 (eMethods in the Supplement).

### Statistical Analysis

First, we assessed differences in characteristics between Black and White enrollees. Second, using a linear model we estimated differences in health care spending and utilization between Black and White enrollees as stratified by children and adults. For each outcome (*Y*) for each enrollee (*i*) we fit *Y_i_* = α + *X_i_* + *HCC_i_* + *Provider_i_* + *βBlack_i_* + *ϵ_i_* in which α was a constant, *X_i_* was a vector of individual-level adjusters (including sex, 5-year age buckets, Medicaid eligibility category, and zip code), *Black_i_* was a variable for whether the enrollee identified as Black, and *ϵ_i_* was noise. In some specifications, we adjusted for enrollee health status (*HCC_i_*,), a vector of 141 Health and Human Services Hierarchical Condition Category indicators, and enrollees’ usual source of care (*Provider_i_*), a vector of fixed effects for the usual source of care to which each enrollee was attributed. The coefficient of interest, β, measured the mean difference in an outcome between Black and White enrollees, adjusting for the other variables in the model. Because annual spending is a skewed, limited dependent variable (eFigure 2 in the Supplement), we assessed the robustness of the spending results to winsorizing or log transforming spending, approaches that are common in the literature.^41^

Third, using enrollee-level characteristics (excluding race), we estimated annual health care spending for each enrollee by applying common risk adjustment methods (eMethods in the Supplement). Based on the model of estimated spending (ie, risk score) for the entire population, we categorized enrollees into 1 of 50 quantiles (within each quantile the enrollees had similar levels of estimated spending). We then compared realized health care spending for Black and White enrollees within each quantile to assess whether enrollees with similar risk scores (based on age, sex, and health conditions) had different levels of realized spending by race. Fourth, we conducted exploratory subgroup analyses in which we stratified by state, county-level urbanicity, county-level racial segregation, zip code–level area deprivation, Medicaid eligibility, and health condition.

Statistical analyses were conducted using 2-tailed tests with Huber-White robust SEs to assess statistical significance, which was defined as *P* < .05. To control the false discovery rate within families of independent hypotheses, we used the Benjamini-Hochberg procedure to adjust *P* values (eMethods in the Supplement). Data analyses were performed from January 28, 2021, to October 18, 2021, using Stata, version 16 (StataCorp) and Python, version 3.8 (Python Software Foundation).

## Results

### Study Population

The study population included 1 966 689 adults and children enrolled in Medicaid (mean [SD] age, 20.3 [17.1] years; 1 119 136 [56.9%] female), of whom 867 183 (44.1%) self-identified as non-Hispanic Black and 1 099 506 (55.9%) self-identified as non-Hispanic White. Demographic and health characteristics did not vary substantially between Black and White enrollees, with the exception that Black enrollees were more likely to live in an urban environment (Table 1). The 3 study states generally had similar urbanicity and health insurance coverage patterns compared with the rest of the country, although the study states were more reliant on MMC, and some demographic characteristics differed from national averages (eTables 1 and 4 in the Supplement). In addition, the level of missingness of race information varied from 7.2% to 24.6% across the study states, motivating sensitivity analyses in which we stratified by state to see if the results were qualitatively different in states with higher levels of missingness.

**Table 1.** Study Population

Characteristic	No. (%)		
	Overall population (N = 1 966 689)	Black enrollees (n = 867 183)	White enrollees (n = 1 099 506)
Age, mean (SD), y	20.3 (17.1)	20.1 (16.9)	20.5 (17.3)
Female	1 119 136 (56.9)	501 170 (57.8)	617 966 (56.2)
Male	847 553 (43.1)	366 013 (42.2)	481 540 (43.8)
Enrolled in MMC	1 856 231 (94.4)	806 065 (93.0)	1 050 166 (95.5)
Geographic characteristics			
Urban	1 482 206 (75.4)	745 029 (85.9)	737 177 (67.0)
Residential segregation, value (SD)	49.5 (12.6)	53 (12.0)	47 (12.5)
Area deprivation index, value (SD)	69.2 (16.1)	71 (16.7)	68 (15.6)
Medicaid eligibility category			
Disability	290 996 (14.8)	126 997 (14.6)	163 999 (14.9)
Child	1 141 213 (58.0)	499 738 (57.6)	641 475 (58.3)
Adult	422 917 (21.5)	192 442 (22.2)	230 475 (21.0)
Other	111 563 (5.7)	48 006 (5.5)	63 557 (5.8)
Health conditions^a^			
No. of conditions, value (SD)	0.45 (1.02)	0.42 (0.96)	0.48 (1.06)
Any condition	523 743 (26.6)	222 808 (25.7)	300 935 (27.4)
Asthma/COPD	200 782 (10.2)	91 687 (10.6)	109 095 (9.9)
Cardiovascular conditions	55 901 (2.8)	22 758 (2.6)	33 143 (3.0)
Depressive/bipolar/psychotic disorders	119 594 (6.1)	40 312 (4.6)	79 282 (7.2)
Diabetes	76 415 (3.9)	35 104 (4.0)	41 311 (3.8)
Drug/substance use disorders	48 449 (2.5)	13 074 (1.5)	35 375 (3.2)
Pregnancy	77 716 (4.0)	36 029 (4.2)	41 687 (3.8)
Seizures	43 436 (2.2)	15 420 (1.8)	28 016 (2.5)

Abbreviations: COPD, chronic obstructive pulmonary disease; HHS-HCC, Health and Human Services Hierarchical Condition Category; MMC, Medicaid managed care.

^a^ Based on HHS-HCCs. For the purposes of reporting, individual HHS-HCCs are grouped into similar descriptive categories: asthma/COPD (HCC 160, 161.1, 161.2), cardiovascular conditions (HCC 125, 126, 127, 128, 129, 130, 131, 132, 135, 137, 138, 139, 142), depressive/bipolar/psychotic disorders (HCC 87.1, 87.2, 88, 90, 102, 103), diabetes (HCC 20, 21) drug/substance use disorders (HCC 81, 82, 83, 84), pregnancy (HCC 203, 204, 205, 207, 208, 209, 210, 211, 212), and seizures (HCC 120). State-specific study population tables are available in eTable 4 in the Supplement.

### Racial Differences in Health Care Spending and Utilization

The study results indicated statistically and economically significant differences in health care spending between Black and White enrollees among children and adults. Annually, spending on adult Black enrollees was $620 (95% CI, $538-$703), or 12%, less than adult White enrollees after adjustment for demographic characteristics (Table 2). Additional adjustments for enrollee health status reduced the magnitude of the difference to $413 (95% CI, $342-$483), a 33% reduction, but the difference remained substantial and statistically significant (Table 2). Results were qualitatively similar for Medicaid children, although the level of the spending differences between Black and White enrollees, in percentage terms, were larger (Table 3). Pooling children and adults, we found that for nearly every decile of estimated enrollee spending, Black enrollees had statistically significantly lower realized spending than White enrollees (Figure). This pattern held when assessing children and adults separately (eFigure 4 in the Supplement).

**Table 2.** Racial Disparities in Health Care Spending and Utilization for Medicaid Adults

Characteristic	Unadjusted means (enrollee)		Total adult population (N = 780 443)			
			Gap between Black and White individuals (95% CI)^a^			*P* value (adjusted)
	Black (n = 344 023)	White (n = 436 420)	Adjusted for demographic characteristics	Adjusted for demographic characteristics and health status	Adjusted for demographic characteristics, health status, and usual source of care	
Health care spending						
Any spending, %	75.87	77.69	0.54 (0.30 to 0.78)	1.57 (1.34 to 1.79)	−0.62 (−0.77 to −0.48)	<.001 (<.001)
Total spending per enrollee per y, $	4356	4998	−620 (−703 to −538)	−413 (−483 to −342)	−317 (−375 to −259)	<.001 (<.001)
Medical spending, $	3330	3586	−389 (−458 to −319)	−178 (−239 to −118)	−92 (−137 to −46)	<.001 (<.001)
Drug spending, $	1026	1412	−232 (−270 to −194)	−234 (−268 to −201)	−225 (−259 to −191)	<.001 (<.001)
Medical care utilization						
Primary care visits, per 100 enrollees per year	358.4	449.3	−41.98 (−45.23 to −38.73)	−17.29 (−20.03 to −14.54)	−19.31 (−21.84 to −16.78)	<.001 (<.001)
Any primary care in a year, %	61.6	65.1	−0.33 (−0.60 to −0.06)	0.87 (0.62 to 1.12)	−0.44 (−0.62 to −0.26)	<.001 (<.001)
Office-based specialty care per 100 enrollees per y	112.8	191.6	−26.23 (−29.46 to −23.01)	−16.27 (−19.47 to −13.06)	−7.19 (−10.06 to −4.33)	<.001 (<.001)
Inpatient hospitalizations per 100 enrollees per y	14.6	14.7	−2.05 (−2.41 to −1.68)	−0.77 (−1.04 to −0.50)	−0.59 (−0.86 to −0.31)	<.001 (<.001)
Laboratory tests and imaging per 100 enrollees per y	208.4	253.5	−15.01 (−17.70 to −12.32)	−0.92 (−2.85 to 1.02)	−3.87 (−5.80 to −1.94)	<.001 (<.001)
ED services per 100 enrollees per y	116.5	113.5	7.77 (6.17 to 9.36)	16.05 (14.61 to 17.48)	9.49 (8.07 to 10.90)	<.001 (<.001)
Select Rx drug utilization, No. of prescriptions per 100 enrollees per y						
All prescriptions	1445.87	1997.8	−372.30 (−389.73 to −354.86)	−317.07 (−331.74 to −302.39)	−315.63 (−330.02 to −301.24)	<.001 (<.001)
Antihypertensives^b^	779.5 (n = 86 666)	746.5 (n = 91 591)	102.28 (91.13 to 113.42)	82.33 (71.24 to 93.43)	86.53 (75.07 to 97.99)	<.001 (<.001)
Asthma medication^b^	422.4 (n = 31 224)	484.4 (n = 56 440)	−50.28 (−64.14 to −36.43)	−54.73 (−68.69 to −40.78)	−58.23 (−72.83 to −43.62)	<.001 (<.001)
Diabetes medication^b^	627.9 (n = 33 303)	764.6 (n = 39 129)	−110.18 (−128.88 to −91.48)	−112.51 (−130.89 to −94.13)	−117.49 (−136.79 to −98.19)	<.001 (<.001)
Statins^b^	226.2 (n = 16 500)	228.2 (n = 23 881)	−24.32 (−34.56 to −14.08)	−45.48 (−55.67 to −35.29)	−48.20 (−59.33 to −37.08)	<.001 (<.001)

Abbreviations: ATC, Anatomical Therapeutic Chemical Classification; ED, emergency department; HHS-HCC, Health and Human Services Hierarchical Condition Category; Rx, prescription.

^a^ Demographic characteristic–adjusted differences included controls for sex, 5-year age buckets, Medicaid eligibility category, and zip code. Health status adjustment added the 141 HHS-HCC indicators as controls. To control the false discovery rate within families of independent hypotheses, we used the Benjamini-Hochberg procedure to adjust *P* values (eMethods in the Supplement).

^b^ Drug groupings were defined using different levels of the ATC system. Antihypertensives were defined by ATC level 2 C02, C03, C07, C08, and C09 and excluded ATC C02KX01, C03BA08, C03CA01, C07AA07, and C07AA12; asthma medications were defined by ATC level 2 R03; diabetes medications were defined by ATC level 2 A10; and statins were defined by ATC level 4 C10AA. Measures were assessed for enrollees with associated diagnosed conditions (eMethods in the Supplement). For measures based on a subset of the population, sample sizes are presented under unadjusted means.

**Table 3.** Racial Disparities in Health Care Spending and Utilization for Children Enrolled in Medicaid

Characteristic	Unadjusted means (enrollee)		Total child population (N = 1 186 246)			
			Gap between Black and White children (95% CI)^a^			*P* value (adjusted)
	Black (n = 523 160)	White (n = 663 086)	Adjusted for demographic characteristics	Adjusted for demographic characteristics and health status	Adjusted for demographic characteristics, health status, and usual source of care	
Health care spending						
Any spending, %	90.84	92.50	−0.63 (−0.76 to −0.50)	−0.78 (−0.91 to −0.65)	−0.35 (−0.40 to −0.29)	<.001 (<.001)
Total spending per enrollee per y, $	$1573	$1848	-$518 (-$561 to -$475)	-$293 (-$328 to -$259)	-$256 (-$290 to -$222)	<.001 (<.001)
Medical spending, $	$1260	$1316	-$284 (-$314 to -$253)	-$107 (-$133 to -$81)	-$94 (-$118 to -$71)	<.001 (<.001)
Drug spending, $	$313	$532	-$234 (-$262 to -$207)	-$186 (-$208 to -$164)	-$162 (-$185 to -$138)	<.001 (<.001)
Medical care utilization						
Primary care visits per 100 enrollees per y	304.7	397.8	−111.25 (−113.27 to −109.22)	−111.79 (−113.69 to −109.89)	−90.06 (−91.80 to −88.31)	<.001 (<.001)
Any primary care in a year, %	78.7	80.7	−3.31 (−3.50 to −3.12)	−3.59 (−3.77 to −3.40)	−1.37 (−1.50 to −1.23)	<.001 (<.001)
Office-based specialty care per 100 enrollees per y	150.1	201.6	−39.57 (−42.08 to −37.06)	−31.37 (−33.71 to −29.04)	−18.83 (−20.55 to −17.11)	<.001 (<.001)
Inpatient hospitalizations per 100 enrollees per y	2.7	2.6	−0.76 (−0.88 to −0.64)	−0.24 (−0.34 to −0.14)	−0.25 (−0.35 to −0.15)	<.001 (<.001)
Laboratory tests and imaging per 100 enrollees per y	104.8	113.0	−13.77 (−14.67 to −12.86)	−13.68 (−14.49 to −12.87)	−11.81 (−12.60 to −11.01)	<.001 (<.001)
ED services per 100 enrollees per y	66.6	61.0	6.07 (5.50 to 6.65)	4.79 (4.24 to 5.34)	2.53 (2.00 to 3.06)	<.001 (<.001)
Select Rx drug utilization, No. of prescriptions per 100 enrollees per y						
All prescriptions	519.81	741.8	−219.45 (−224.76 to −214.13)	−219.49 (−224.19 to −214.78)	−185.76 (−190.33 to −181.18)	<.001 (<.001)
Asthma medication^b^	468.6 (n = 60 463)	541.3 (n = 52 655)	−21.79 (−31.62 to −11.96)	−19.47 (−29.19 to −9.75)	−10.66 (−20.70 to −0.61)	.04 (.08)
Diabetes medication^b^	559.2 (n = 1801)	822.2 (n = 2182)	−152.09 (−225.24 to −78.93)	−47.81 (−110.83 to 15.21)	−65.44 (−152.20 to 21.32)	.14 (.16)

Abbreviations: ATC, Anatomical Therapeutic Chemical Classification; ED, emergency department; HHS-HCC, Health and Human Services Hierarchical Condition Category.

^a^ Demographic characteristic–adjusted differences included controls for sex, 5-year age buckets, Medicaid eligibility category, and zip code. Health status adjustment added the 141 HHS-HCC indicators as controls. To control the false discovery rate within families of independent hypotheses, we used the Benjamini-Hochberg procedure to adjust *P* values (eMethods in the Supplement).

^b^ Drug groupings were defined using different levels of the ATC system. Asthma medications were defined by ATC level 2 R03, and diabetes medications were defined by ATC level 2 A10. Measures were assessed for enrollees with associated diagnosed conditions (eMethods in the Supplement). For measures based on a subset of the population, sample sizes were presented under unadjusted means.

**Figure.**
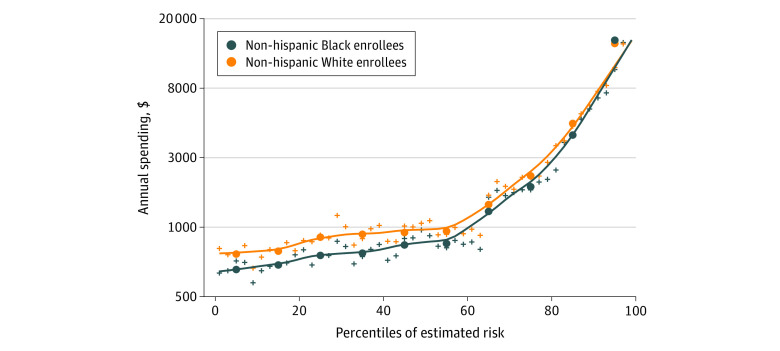
Annual Health Care Spending by Percentile of Estimated Risk and Race Total annual health care spending by race as a function of risk score (ie, estimated spending). Estimated risk is based on a full-sample, concurrent estimation of spending that uses enrollees’ age in years, Medicaid eligibility category, sex, and the 141 Health and Human Services Hierarchical Condition Category indicators. The plus signs show the 50 quantiles, and dots indicate deciles. We plotted 95% CIs for each decile, but they are not visible at this scale because of the precision of the estimates. Quantiles are based on the estimated risk for the entire population pooling across race. The y-axis is truncated at the top for visibility. An untruncated version is available in eFigure 3 in the Supplement.

Consistent with lower spending, Black enrollees generally utilized fewer medical services than White enrollees after adjusting for demographic characteristics and health status. For example, adult Black enrollees had 17.3 (95% CI, 14.5-20.0) fewer primary care encounters per 100 enrollees per year compared with adult White enrollees, a 4% difference, despite being 0.9 percentage points (95% CI, 0.6-1.1) more likely to use any primary care in a year (Table 2). Among children the differences were larger: Black enrollees had 111.8 (95% CI, 109.9-113.7) fewer primary care encounters per 100 enrollees per year compared with White enrollees, a 28% difference, and were 3.6 percentage points (95% CI, 3.4-3.8) less likely to use any primary care in a year (Table 3). Among children and adults, Black enrollees utilized fewer of the other categories of medical services we examined except for the emergency department, for which Black adults had 16.1 (14%) more emergency department visits than White adults (95% CI, 14.6-17.5), and Black children had 4.8 (8%) more emergency department visits than White children (Table 2 and Table 3).

Black enrollees also filled fewer prescription drugs than White enrollees, although patterns differed by therapeutic class. After adjustment for demographic characteristics and health status, adult Black enrollees filled 317.1 (16%) fewer prescriptions per 100 enrollees annually (95% CI, 302.4-331.7) than adult White enrollees, and Black enrollees with asthma, diabetes, and cardiovascular conditions were less likely to fill prescriptions for asthma medication, diabetes medication, and statins, respectively. However, adult Black enrollees filled more prescriptions for antihypertensives than White enrollees (Table 2). Compared with White children in Medicaid, Black children enrolled in Medicaid also filled fewer prescription drugs overall, and those with asthma or diabetes filled fewer prescriptions for asthma and diabetes medication (Table 3; eTable 5 in the Supplement).

Additionally adjusting for enrollees’ usual source of care reduced the racial difference in total spending by 23% for adults and 13% for children, suggesting that one-eighth to one-fifth of the observed differences in spending were explained by differences in practice patterns of health care professionals or medical institutions utilized by Black and White enrollees. Adjusting for differences in practice patterns generally reduced differences for other health care spending and utilization outcomes, although the magnitudes of the reduction differed. Differences in spending remained substantial in robustness analyses (eTable 6 in the Supplement).

### Racial Differences in Preventive Care and Care of Acute and Chronic Conditions

Despite Black enrollees utilizing fewer services overall and less primary care, they had higher rates of HEDIS preventive care screening measures after adjusting for demographic characteristics and health status (Table 4). For example, compared with adult White enrollees, adult Black enrollees were 5.4 percentage points (17%) more likely to receive a breast cancer screening (95% CI, 4.6-6.1), 7.8 percentage points (21%) more likely to receive a cervical cancer screening (95% CI, 7.4-8.3), and 13.5 percentage points (29%) more likely to get screened for chlamydia (95% CI, 12.4-14.6). Rates of HEDIS preventive care measures were also higher among Black children than White children enrolled in Medicaid. Black children were 5.1 percentage points (11%) more likely to have an annual well-child visit (95% CI:4.8-5.5) and 8.5 percentage points (22%) more likely to get a screening for chlamydia (95% CI:7.1-9.9). Adjusting for enrollees’ usual source of care attenuated, but did not eliminate, those differences.

**Table 4.** Racial Disparities in Preventive Care and Care of Acute and Chronic Conditions^a^

Characteristic	Unadjusted means (enrollee)		Gap between Black and White individuals (95% CI)		Adjusted for demographic characteristics, health status, and usual source of care	
	Black	White	Adjusted for demographic characteristics	Adjusted for demographic characteristics and health status	Gap between Black and White individuals (95% CI)	*P* value (adjusted)
**Panel 1: adults**						
No.	344 023	436 420	780 443	780 443	780 443	NA
Preventive care						
Breast cancer screening^b^	34.8 (n = 39 890)	31.1 (n = 51 014)	5.27 (4.48 to 6.06)	5.39 (4.64 to 6.14)	4.06 (3.31 to 4.81)	<.001 (<.001)
Cervical cancer screening^b^	45.9 (n = 132 294)	37.1 (n = 90 650)	7.85 (7.39 to 8.30)	7.80 (7.35 to 8.25)	5.72 (5.27 to 6.16)	<.001 (<.001)
Chlamydia screening^b^	68.4 (n = 27 168)	46.8 (n = 19 861)	12.82 (11.69 to 13.95)	13.48 (12.37 to 14.60)	9.65 (8.52 to 10.78)	<.001 (<.001)
Care of acute and chronic conditions						
Pharmacotherapy for opioid use disorder^b^	12.0 (n = 3504)	28.6 (n = 18 376)	−15.17 (−16.83 to −13.52)	−13.92 (−15.62 to −12.21)	−8.84 (−10.63 to −7.05)	<.001 (<.001)
Comprehensive diabetes care: hemoglobin A_1c_ testing^b^	56.5 (n = 32 153)	48.3 (n = 37 305)	−0.85 (−1.76 to 0.06)	−0.84 (−1.70 to 0.02)	−0.53 (−1.40 to 0.34)	.23 (.39)
Diabetes screening for people with schizophrenia^b^	66.9 (n = 9204)	68.5 (n = 14 303)	−2.73 (−4.25 to −1.22)	−0.12 (−1.65 to 1.40)	−0.13 (−1.76 to 1.51)	.88 (.88)
Asthma medication ratio^b^	38.9 (n = 4234)	40.2 (n = 5331)	0.01 (−2.88 to 2.90)	−0.57 (−3.52 to 2.37)	−0.69 (−4.26 to 2.88)	.71 (.88)
Potentially avoidable ED visits per 100 enrollees per y	29.5	26.1	4.07 (3.56 to 4.57)	5.95 (5.47 to 6.43)	4.29 (3.81 to 4.77)	<.001 (<.001)
**Panel 2: children**						
No.	523 160	663 086	1 186 246	1 186 246	1 186 246	NA
Preventive care						
Annual well-child visits^b^	55.4 (n = 308 087)	47.9 (n = 388 887)	5.36 (5.05 to 5.67)	5.14 (4.83 to 5.46)	5.16 (4.87 to 5.45)	<.001 (<.001)
Chlamydia screening^b^	58.0 (n = 15 562)	38.9 (n = 14 948)	8.72 (7.30 to 10.14)	8.49 (7.09 to 9.89)	5.45 (3.99 to 6.91)	<.001 (<.001)
Care of acute and chronic conditions						
Asthma medication ratio^b^	67.7 (n = 13 478)	72.9 (n = 14 050)	−1.60 (−3.07 to −0.13)	−1.49 (−2.97 to −0.01)	−1.18 (−2.79 to 0.44)	.15 (.15)
Potentially avoidable ED visits per 100 enrollees per y	16.0	12.6	3.57 (3.35 to 3.79)	3.07 (2.85 to 3.29)	2.33 (2.11 to 2.55)	<.001 (<.001)

Abbreviations: ED, emergency department; HEDIS, Healthcare Effectiveness Data and Information Set; HHS-HCC, Health and Human Services Hierarchical Condition Category; NA, not applicable.

^a^ Demographic characteristic–adjusted differences included controls for sex, 5-year age buckets, Medicaid eligibility category, and zip code. Health status adjustment added the 141 HHS-HCC indicators as controls. To control the false discovery rate within families of independent hypotheses, we used the Benjamini-Hochberg procedure to adjust *P* values (eMethods in the Supplement).

^b^ Based on HEDIS specification. Units are the share of the qualifying population that adheres to the HEDIS recommendation. Regressions were only among enrollees qualifying for the denominator of each measure (based on age, sex, or disease-related inclusion or exclusion criteria specific to that measure). For measures based on a subset of the population, sample sizes are presented under unadjusted means.

By comparison, Black enrollees either utilized less recommended care for acute and chronic conditions or there were no racial differences (Table 4). For example, among adults, Black enrollees were 13.9 percentage points (48%) less likely to receive treatment with pharmacotherapy for opioid use disorder (95% CI, 12.2-15.6) than White enrollees after adjustment for demographic characteristics and health status. However, we did not detect statistically significant racial differences in HEDIS measures for hemoglobin A_1c_ testing, diabetes screening for people with schizophrenia, or asthma medication ratios. Adult Black enrollees had 5.95 (23%) more emergency department visits for avoidable reasons per 100 enrollees per year compared with adult White enrollees (95% CI, 5.47-6.43) even after adjustment for demographic characteristics and health status. Among children, Black enrollees had more emergency department visits for avoidable reasons compared with adult White enrollees and were less likely to receive recommended care for asthma. Adjusting for enrollees’ usual source of care attenuated, but generally did not eliminate, racial differences.^42,43,44,45^ One exception to this was children’s receipt of recommended asthma medication, for which there was no longer a statistically significant racial difference after adjustment. Patterns of racial differences in health care spending, primary care, and avoidable emergency department were found to be broadly consistent in exploratory analyses that stratified by state, geography, Medicaid eligibility, and health status (eFigures 5-7 in the Supplement).

## Discussion

In this cross-sectional, multistate study of nearly 2 million Medicaid enrollees in 2016, Black enrollees generated lower spending and used fewer services, including recommended care for acute and chronic conditions, but had substantially higher emergency department use. These differences remained large after adjusting for enrollee-level confounders and persisted when making comparisons between enrollees who were treated by the same health care professionals or medical institutions. Despite lower utilization, Black enrollees had higher rates of HEDIS preventive screenings than White enrollees. These findings were broadly consistent for adults and children and across all 3 of the study states, in rural and urban regions, and across zip codes that varied by residential racial segregation and socioeconomic deprivation.

Many states expanded Medicaid to cover remaining uninsured individuals, with hopes that this would increase access to care and reduce health inequalities. These expansions reduced health disparities,^13^ but the results of this study suggest that coverage alone does not eliminate racial disparities. While racial differences in health care service do not always imply a disparity (because distinct groups have different needs, perceptions, and experiences that shape their demand for care^3^) it is important to put this study’s findings in the context of historical concerns about access in Medicaid^46,47^ and the goal of expanding access to primary care as a key motivation for adopting managed care.^48,49^ In addition, it is well-documented that racial and ethnic minority groups face structural and interpersonal racism that harm their health and reduce access to care.^25,28,42,44,50^ In this context, lower utilization of primary care suggests that Black enrollees are underserved rather than there being overuse by White enrollees. Black and White enrollees initiated care at similar rates (ie, there were small racial differences in the likelihood of using any primary care), implying that racial differences in primary care (and other) utilization tended to emerge after care was initiated, which may be consistent with evidence that even when access barriers are overcome, Black patients receive worse care and experience the health care system differently^51,52,53^ as a result of medical racism,^54^ discrimination by health care professionals,^43,49,55^ and differences in how physicians perceive them.^25,26,27,45,56,57^ For example, we found that Black adults were 48% less likely to receive treatment with pharmacotherapy for opioid use disorders, which was consistent with prior literature showing racialized access to these medications.^28,58^ While racial differences in the quality measures were nuanced (eg, Black enrollees had higher rates of preventive screenings but lower utilization of care for acute and chronic conditions), Black adults and children had higher emergency department utilization, including for avoidable reasons, reinforcing the idea that disparities in primary care reflect underuse.

The results of this study also have implications for how to promote health equity in Medicaid. When we stratified by risk score, Black enrollees had lower realized spending than White enrollees with the same risk scores. These findings have implications for MMC policy. First, lower realized (compared with estimated) spending for Black enrollees suggests that improving the prediction of risk adjustment systems for underserved groups could reduce risk-adjusted prospective payments to plans serving those populations, reinforcing current spending deficits.^59^ Second, because risk adjustment models are calibrated using current spending levels, lower spending as a result of unmet need for racial and ethnic minority groups is associated with undercompensation for health conditions that are prevalent in these groups. Rather than relying on current spending levels to set risk adjustment weights, policy makers should consider first transforming health care spending to desired levels.^60^ In addition to these implications for risk adjustment, this study’s results suggest the need to align the incentives of MMC plans and health care professionals around better understanding and addressing health equity.

### Limitations

This study has several limitations. First, data limitations in the coding of race and ethnicity and sample size issues limited us to comparing non-Hispanic Black and non-Hispanic White enrollees. Whether similar differences exist between other racial and ethnic minority groups in Medicaid is unclear and warrants additional investigation. In addition, there was missingness in the race information we obtained from the study states; systematic differences by race in the types of enrollees with missing information could bias the study results. However, results were similar when we stratified by state despite the differences in rates of missingness.

Second, adjusting for health status is complicated by the fact that measures of health rely on diagnoses and procedures from claims data. Because Black enrollees use fewer services than White enrollees, they are less likely to have administrative claim records. As a result, Black enrollees have fewer documented health conditions than White enrollees with the same underlying health status; hence, the study’s risk-adjusted estimates are a lower bound on the true differences in spending and utilization between Black and White enrollees.

Third, 2 of the 3 study states are geographically concentrated in the South, which has a unique historical racialized institutional context^61^ and relies heavily on MMC. Hence, the study findings may not generalize to other Medicaid programs, particularly in states that primarily operate via fee for service and have different racial and ethnic histories. In addition, the measures associated with health care utilization, preventive care, and care of acute and chronic conditions, while comprehensive, were not complete. Racial differences may differ for other measures. Finally, because the study was conducted during a single year, it is unclear whether disparities are improving (or worsening).

## Conclusions

In this cross-sectional study of US Medicaid enrollees in 3 states, Black enrollees generated lower spending and used fewer services, including recommended care for acute and chronic conditions, but had substantially higher emergency department use. While Black enrollees had higher rates of HEDIS preventive screenings, the study results suggest that additional efforts are needed to understand and promote equitable access in Medicaid.

## Notes

Supplement. **eMethods.** Supplemental description of methods **eFigure 1.** Flow diagram of sample exclusions to arrive at the study population **eFigure 2.** Distribution of all health care spending **eFigure 3.** Annual health care spending by quantile of estimated risk and race without truncation **eFigure 4.** Annual health care spending by quantile of estimated risk and race, stratified by adults and children **eFigure 5.** Racial Differences Between non-Hispanic Black and non-Hispanic White Enrollees in Total Spending, Primary Care Utilization, and Avoidable Emergency Department Utilization, by Subgroup **eFigure 6.** Racial Differences Between non-Hispanic Black and non-Hispanic White Women in Medicaid in Total Spending, Primary Care Utilization, and Avoidable Emergency Department Utilization, by Subgroup **eFigure 7.** Racial Differences Between non-Hispanic Black and non-Hispanic White Men in Medicaid in Total Spending, Primary Care Utilization, and Avoidable Emergency Department Utilization, by Subgroup **eTable 1.** Sample state characteristics in 2016 **eTable 2.** Self-reported racial and ethnic descriptions by state **eTable 3. **Description of HEDIS Performance Measures **eTable 4. **Study population by state **eTable 5. **Additional prescription drug quantity measures, including any fills within a year and medication possession ratio **eTable 6.** Robustness of racial disparities to transformations of health care spending
