# Supplementary material for: Disparities in Health Care Spending and Utilization Among Black and White Medicaid Enrollees
Source: JAMA Health Forum. 2022 Jun 10;3(6):e221398. doi: 10.1001/jamahealthforum.2022.1398 (PMC9187949; doi:10.1001/jamahealthforum.2022.1398)
Supplement: Supplement. — eMethods. Supplemental description of methods eFigure 1. Flow diagram of sample exclusions to arrive at the study population eFigure 2. Distribution of all health care spending eFigure 3. Annual health care spending by quantile of estimated risk and race without truncation eFigure 4. Annual health care spending by quantile of estimated risk and race, stratified by adults and children eFigure 5. Racial Differences Between non-Hispanic Black and non-Hispanic White Enrollees in Total Spending, Primary Care Utilization, and Avoidable Emergency Department Utilization, by Subgroup eFigure 6. Racial Differences Between non-Hispanic Black and non-Hispanic White Women in Medicaid in Total Spending, Primary Care Utilization, and Avoidable Emergency Department Utilization, by Subgroup eFigure 7. Racial Differences Between non-Hispanic Black and non-Hispanic White Men in Medicaid in Total Spending, Primary Care Utilization, and Avoidable Emergency Department Utilization, by Subgroup eTable 1. Sample state characteristics in 2016 eTable 2. Self-reported racial and ethnic descriptions by state eTable 3. Description of HEDIS Performance Measures eTable 4. Study population by state eTable 5. Additional prescription drug quantity measures, including any fills within a year and medication possession ratio eTable 6. Robustness of racial disparities to transformations of health care spending [file jamahealthforum-e221398-s001.pdf]

Supplemental Online Content

Wallace J, Lollo A, Duchowny KA, Lavallee M, Ndumele CD. Disparities in health care spending and utilization among Black and White Medicaid enrollees.2022;3(6):e221398
JAMA Health Forum. doi:10.1001/jamahealthforum.2022.1398

eMethods. Supplemental description of methods
eFigure 1. Flow diagram of sample exclusions to arrive at the study population
eFigure 2. Distribution of all health care spending
eFigure 3. Annual health care spending by quantile of estimated risk and race without truncation
eFigure 4. Annual health care spending by quantile of estimated risk and race, stratified by adults and children
eFigure 5. Racial Differences Between non-Hispanic Black and non-Hispanic White Enrollees in Total Spending, Primary Care Utilization, and Avoidable Emergency Department Utilization, by Subgroup
eFigure 6. Racial Differences Between non-Hispanic Black and non-Hispanic White Women in Medicaid in Total Spending, Primary Care Utilization, and Avoidable Emergency Department Utilization, by Subgroup
eFigure 7. Racial Differences Between non-Hispanic Black and non-Hispanic White Men in Medicaid in Total Spending, Primary Care Utilization, and Avoidable Emergency Department Utilization, by Subgroup
eTable 1. Sample state characteristics in 2016
eTable 2. Self-reported racial and ethnic descriptions by state
eTable 3. Description of HEDIS Performance Measures
eTable 4. Study population by state
eTable 5. Additional prescription drug quantity measures, including any fills within a year and medication possession ratio
eTable 6. Robustness of racial disparities to transformations of health care spending

This supplemental material has been provided by the authors to give readers additional information about their work.

© 2022 Wallace J et al. *JAMA Health Forum.*

Supplementary Appendices

eMethods. Supplemental description of methods

A. Medicaid Eligibility Categorization

Among enrollees younger than 65 years old, we categorize their Medicaid eligibility for 2016 into one of four mutually exclusive categories (disability, child, adult, other) based on their plurality eligibility reason for that year (plurality in the case of different eligibility categories throughout the year). Each state provides state-specific descriptors of their eligibility across two different fields (major eligibility category and specific type case within that category).

Depending upon the state, disability is identified either by its own major eligibility category ("Disability") or specific type cases related to disability ("Aid to Disabled"). As a general rule, disability is defined as enrollees who receive disability related supplemental security income (SSI) or meet other Social Security Administration (SSA) requirements for disability. Child eligibility is determined from and entire major eligibility category (e.g., "Children's Health Insurance Program" (CHIP)) or from major eligibility categories (e.g., "Families and Children", "Parents/Caretake Relative") with an age restriction of the enrollee being 18 years old or younger. Children in these major eligibility categories who would have type cases related to disability would instead be categorized as disabled. Similarly, adults are non-disabled enrollees in these major eligibility categories who are 19 to 64 years old. Any enrollees uncategorized by the above logic are categorized as other, which includes enrollees younger than 65 years who are dually eligible for Medicare, inmates, refugees, enrollees who require long-term care, as well as any state-specific special Medicaid populations.

B. Definition of Racial and Ethnic Collective Terms

In each of the three states used for the analysis racial and ethnic data are self-reported. However, because the exact terms differ across states we define 7 mutually exclusive and completely exhaustive collective terms so that we can pool the analyses across states using common terminology. The 7 collective terms we use are Asian, Black, Hispanic, Native American or Alaska Native, Native Hawaiian or Pacific Islander, White, and Other. In eTable 1, we present the exact descriptions of the self-reported race and ethnicity data as well as the collective term we used for the analyses.

C. HHS-HCC Model

The HHS-HCC model combines demographic and diagnostic information to construct an enrollee level risk score which measures how costly that enrollee is expected to be (in terms of health care expenditures). Instead of constructing a risk score, we use the 141 conditions to generate enrollee-level controls that we add to our regressions. These conditions are hierarchical so that enrollees are ultimately assigned to only the most severe manifestation of their disease as opposed to all manifestations (e.g., an enrollee with diagnoses qualifying for both chronic and acute pancreatitis would be hierarchically categorized with just chronic pancreatitis).

© 2022 Wallace J et al. *JAMA Health Forum.*

D. Usual source of care attribution

Using all paid and denied medical claims in 2016 we attribute each claim to a single usual source of care – the billing provider identified by their National Provider Identifier (NPI). The billing provider field is always present for all claims across all states. For each enrollee we determine the billing provider they received the plurality of their care from based on the largest count of unique claims. In the case of ties, an enrollee was attributed to the health care professional or medical institution that they had a claim with earlier in the year. Enrollees with 0 medical claims could not be attributed to a usual source of care and were instead attributed to a "Missing" usual source of care (unique for each state).

E. Model for estimated risk

Using the full population of enrollees, we generate a concurrent estimation of total health care spending in 2016 using enrollee level characteristics and the 141 HHS-HCC indicators. Total annual health care spending Y for each enrollee i is modeled as:

$$Y_i = \alpha + \beta_1 Age_i + \beta_2 Gender_i + \beta_3 Eligibility_i + \beta_4 HCC_i + \delta_1 State_i + \delta_2 ZIP_i + \epsilon_i$$

where Age_i are 1-year indicators for an enrollee's age, $Gender_i$ are indicators for an enrollee's gender, $Eligibility_i$ are indicators for an enrollee's eligibility category, HCC_i are the 141 HHS-HCC condition indicators for that enrollee, and ϵ_i is a noise term. We also include controls for an enrollee's state $State_i$ and ZIP code of residence ZIP_i to deal with any differences that might arise due to differences in the Medicaid program across states or regions. Enrollee race is not included in this model.

Using the coefficients obtained from the above model, we create our measure of enrollee-level estimated risk $\widehat{Y_i}$ from all of the predictors, except state and ZIP code.

F. Construction of enrollee categories for drug conditioning

To identify enrollees with different health conditions, we use the following logic:

- Asthma: enrollees with HCCs 160, 161.1, 161.2
- Diabetes: enrollees with HCCs 20 and 21
- Cardiovascular condition (used to condition our Statin utilization measures): enrollees with HCCs 125, 126, 127, 128, 129, 130 131, 132, 135, 137, 138, 139, 142
- Hypertension: enrollees with ICD-10 code I10

These health conditions are then used to condition our drug utilization measures and Medication Possession Ratios in Table 2 and eTable 6.

G. Multiple Inference Correction

To adjust for multiple inference, we use the Benjamini-Hochberg procedure to account for testing of multiple outcomes in Tables 2, 3, and 4. To adjust for multiple outcomes we examined within each group of outcomes within each table (and in Table 4, separately within the kids and adults samples), we use the Benjamini-Hochberg procedure to control the false discovery rate at the 5% significance level. We report adjusted *P* values in braces below the unadjusted *P* values in Tables 2, 3, and 4. Additional information on the Benjamini-Hochberg procedure is available elsewhere.[1,2]

© 2022 Wallace J et al. *JAMA Health Forum.*

eFigure 1. Flow diagram of sample exclusions to arrive at the study population

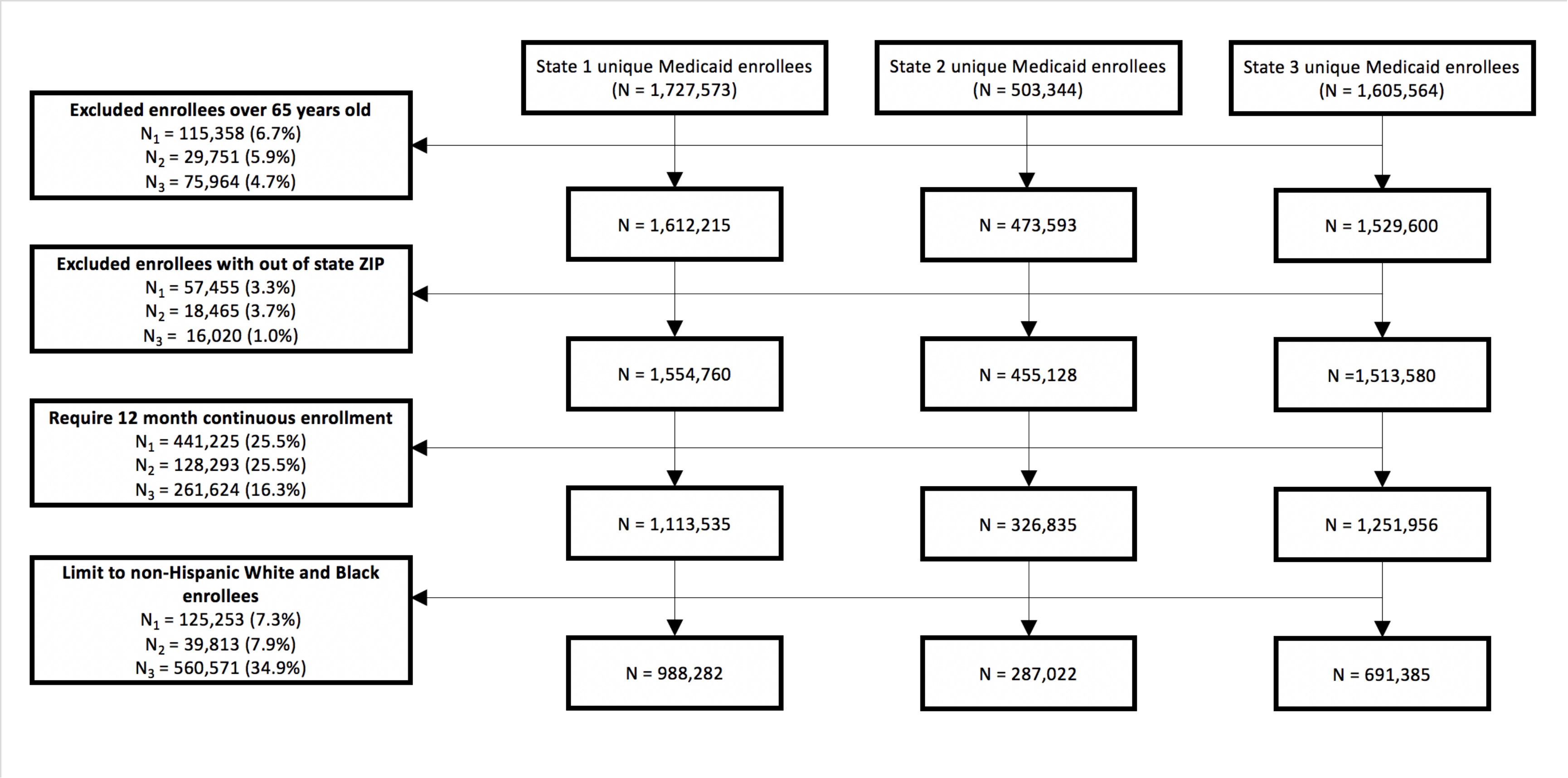

© 2022 Wallace J et al. *JAMA Health Forum.*

eFigure 2. Distribution of all health care spending

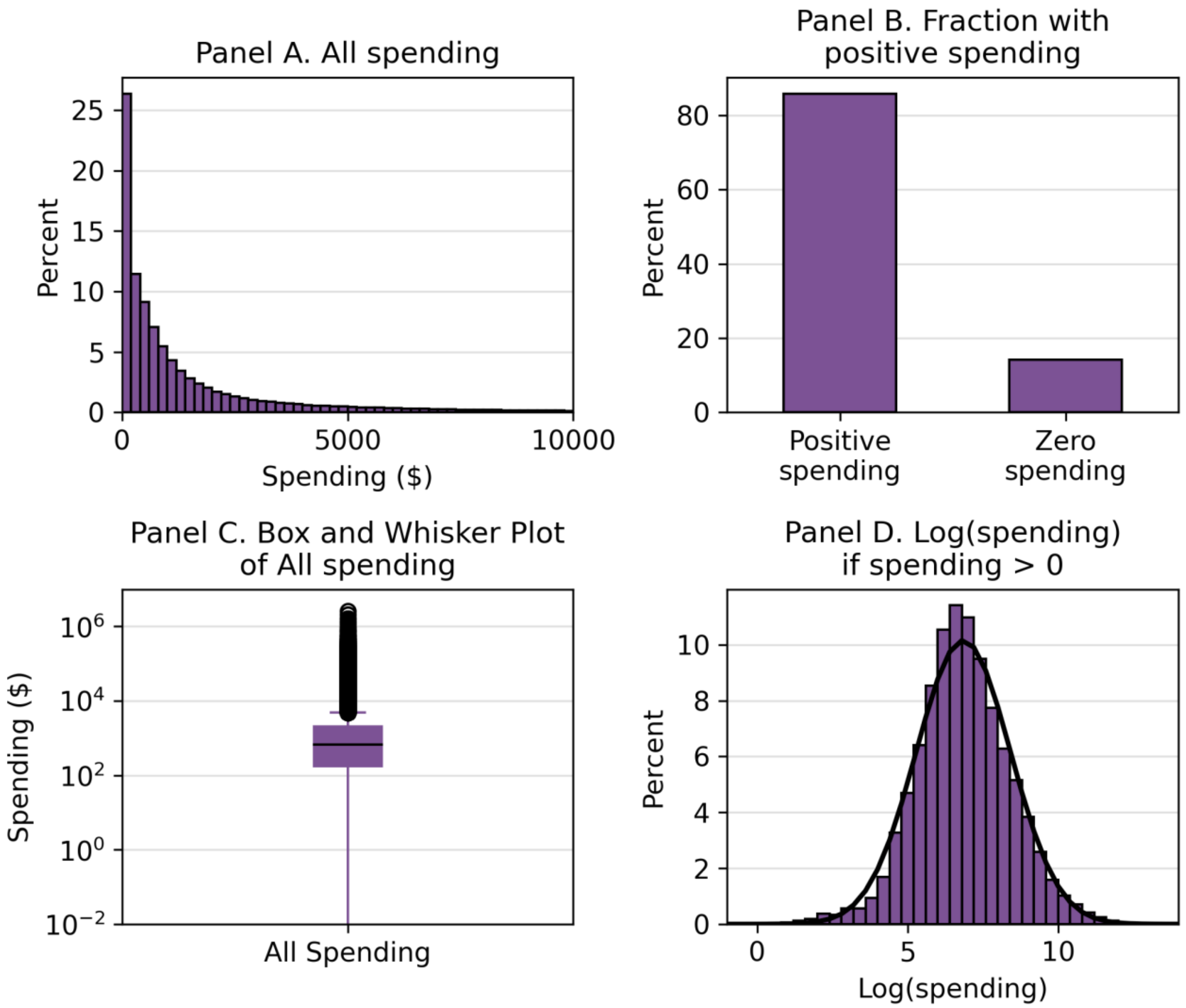

Legend: Distribution of annual total medical and drug spending per enrollee in our study population. Panel A illustrates the distribution of spending, truncated to the right at $10,000 for viewing. Panel B illustrates the percentage of member-years with $0 in annual spending. Panel C shows a box and whisker plot of all spending to highlight the extreme outliers with large spending that were not shown in panel A. Due to the logarithmic scaling of the y-axis and possibility of $0 in annual spending, panel C is clipped below at $0.01. Panel D shows a logarithmic transformation of spending, conditional on positive spending, with a normal distribution overlayed in black.

© 2022 Wallace J et al. *JAMA Health Forum.*

eFigure 3. Annual health care spending by quantile of estimated risk and race without truncation

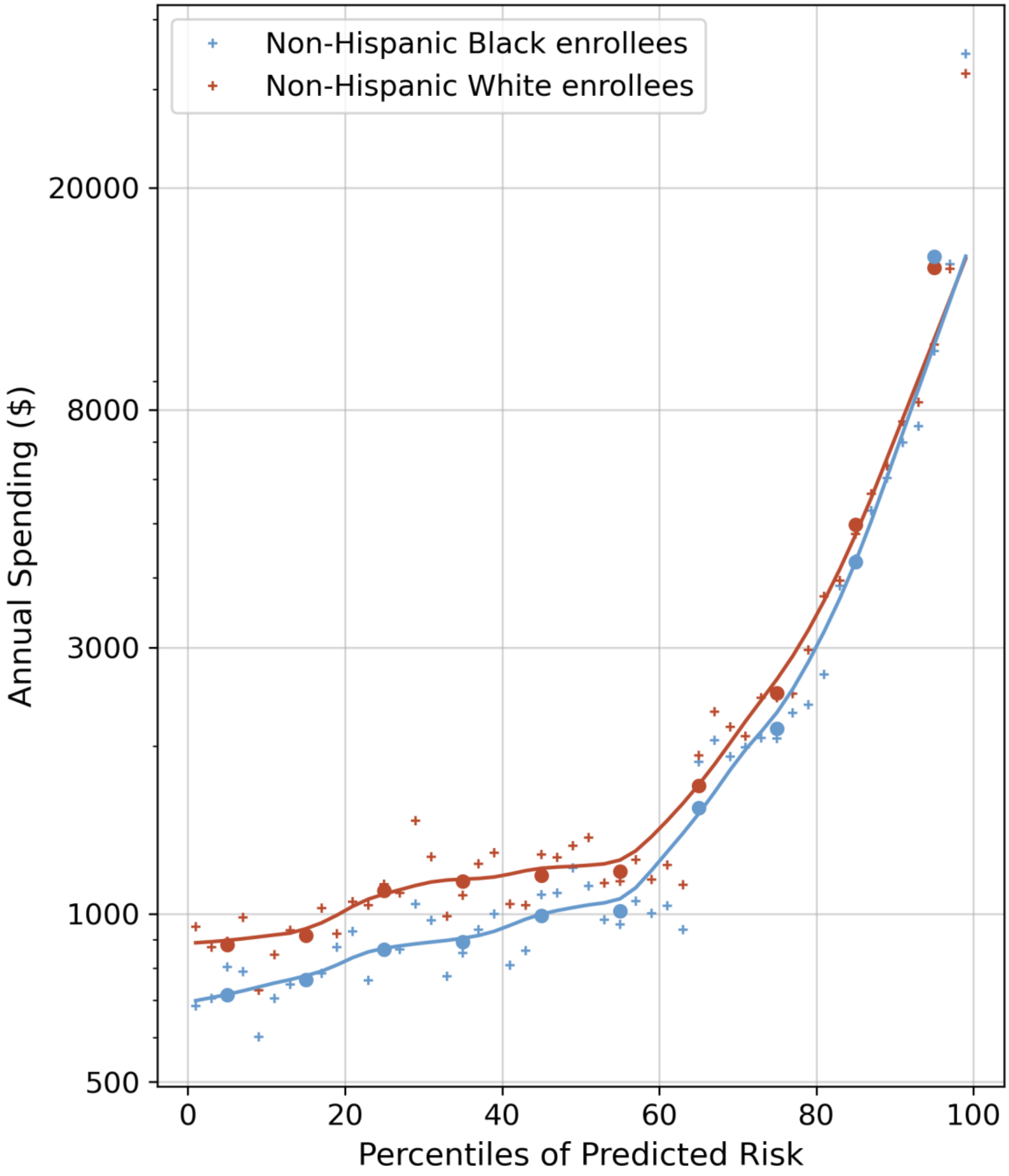

Legend: Total annual health care spending by race as a function of estimated risk. The estimation is a full-population, concurrent estimation based on an enrollee's age in years, Medicaid eligibility category, gender, and the 141 HHS-HCC indicators. + markers show the 50-quantiles, dots indicate deciles. Percentiles are based on the estimated risk in the entire population pooling across race.

© 2022 Wallace J et al. *JAMA Health Forum.*

eFigure 4. Annual health care spending by quantile of estimated risk and race, stratified by adults and children

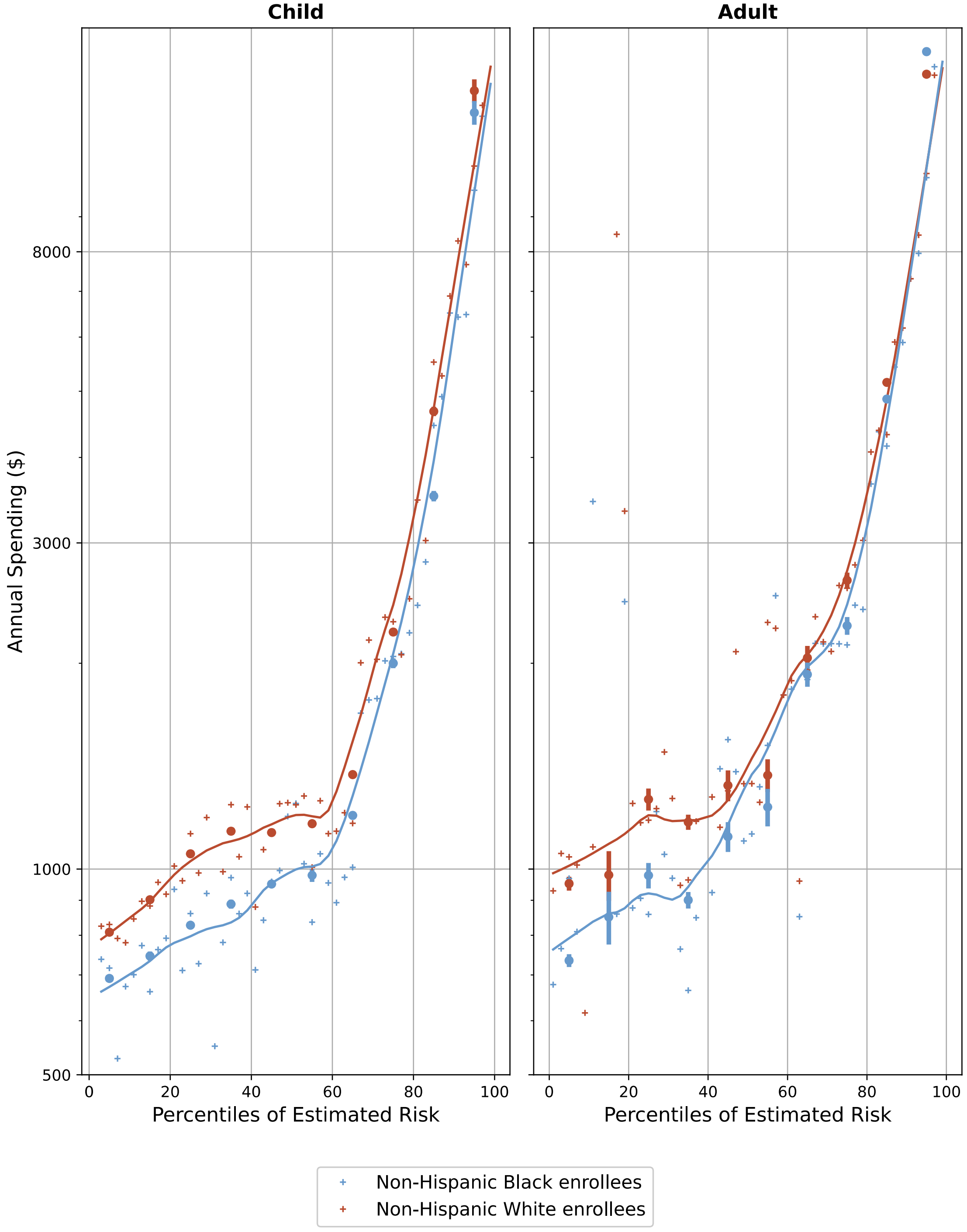

Legend: Total annual health care spending by race as a function of estimated risk. The estimation is a full-population, concurrent estimation based on an enrollee's age in years, Medicaid eligibility category, gender, and the 141 HHS-HCC indicators. + markers show the 50-quantiles, dots indicate deciles. Percentiles are based on the estimated risk in the entire population pooling across race, and then the sample is divided by age where adults are 19 and older.

© 2022 Wallace J et al. *JAMA Health Forum.*

eFigure 5. Racial Differences Between non-Hispanic Black and non-Hispanic White Enrollees in Total Spending, Primary Care Utilization, and Avoidable Emergency Department Utilization, by Subgroup

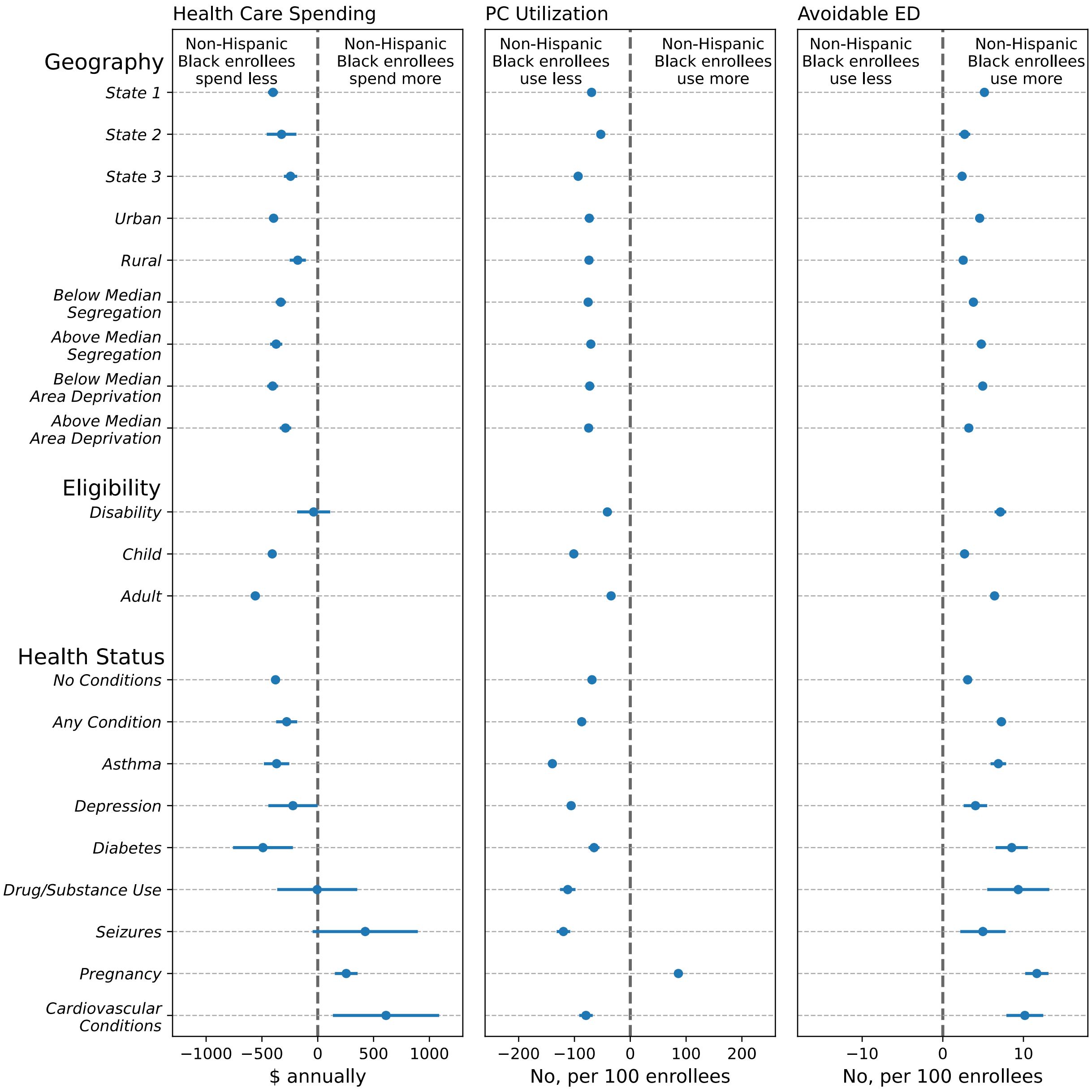

Legend: Estimates and 95% CIs for health care spending, primary care (PC) utilization, and avoidable emergency department (ED) visit rates for non-Hispanic Black enrollees relative to non-Hispanic White enrollees.

© 2022 Wallace J et al. *JAMA Health Forum.*

eFigure 6. Racial Differences Between non-Hispanic Black and non-Hispanic White Women in Medicaid in Total Spending, Primary Care Utilization, and Avoidable Emergency Department Utilization, by Subgroup

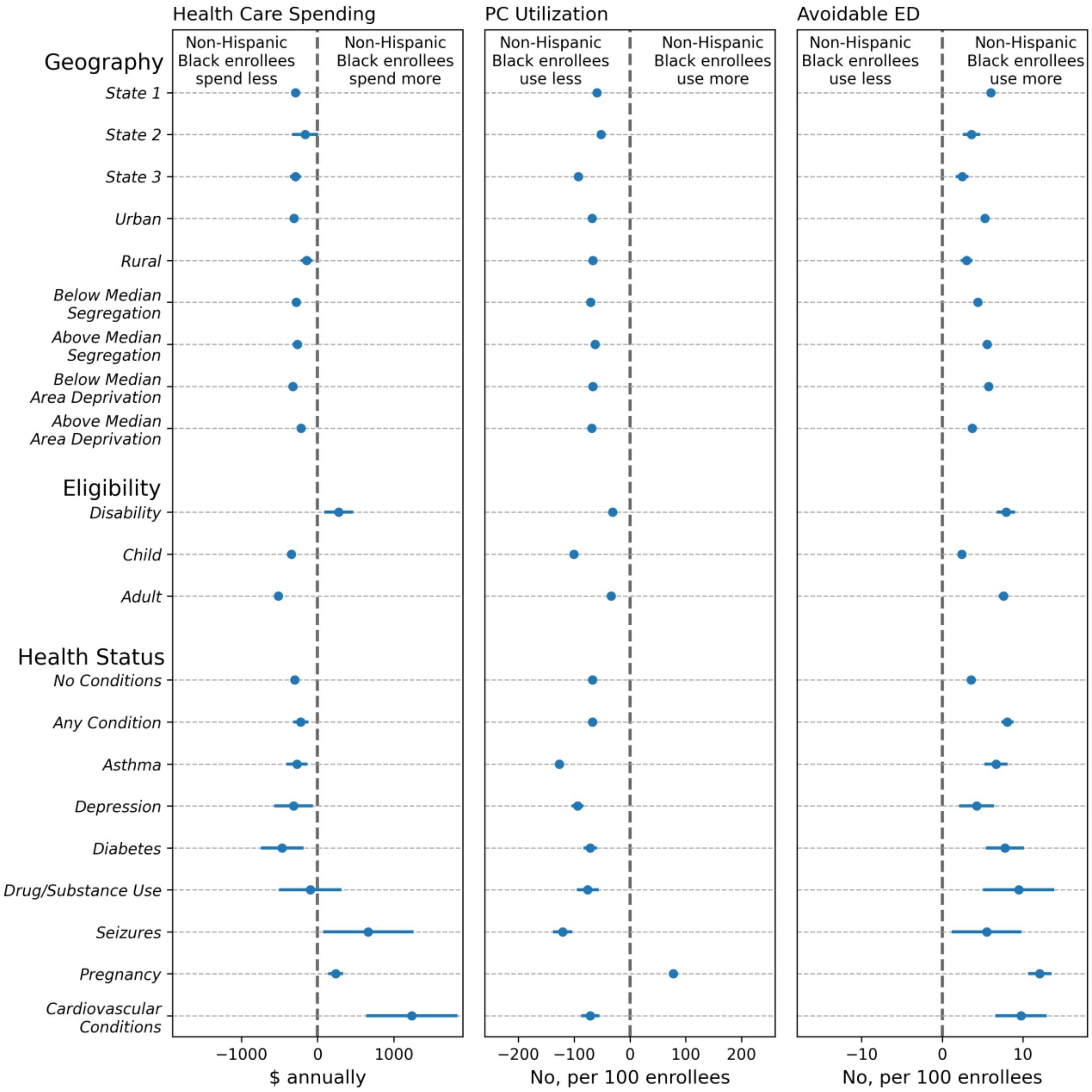

Legend: Estimates and 95% CIs for health care spending, primary care (PC) utilization, and avoidable emergency department (ED) visit rates for non-Hispanic Black women relative to non-Hispanic White women.

© 2022 Wallace J et al. *JAMA Health Forum.*

eFigure 7. Racial Differences Between non-Hispanic Black and non-Hispanic White Men in Medicaid in Total Spending, Primary Care Utilization, and Avoidable Emergency Department Utilization, by Subgroup

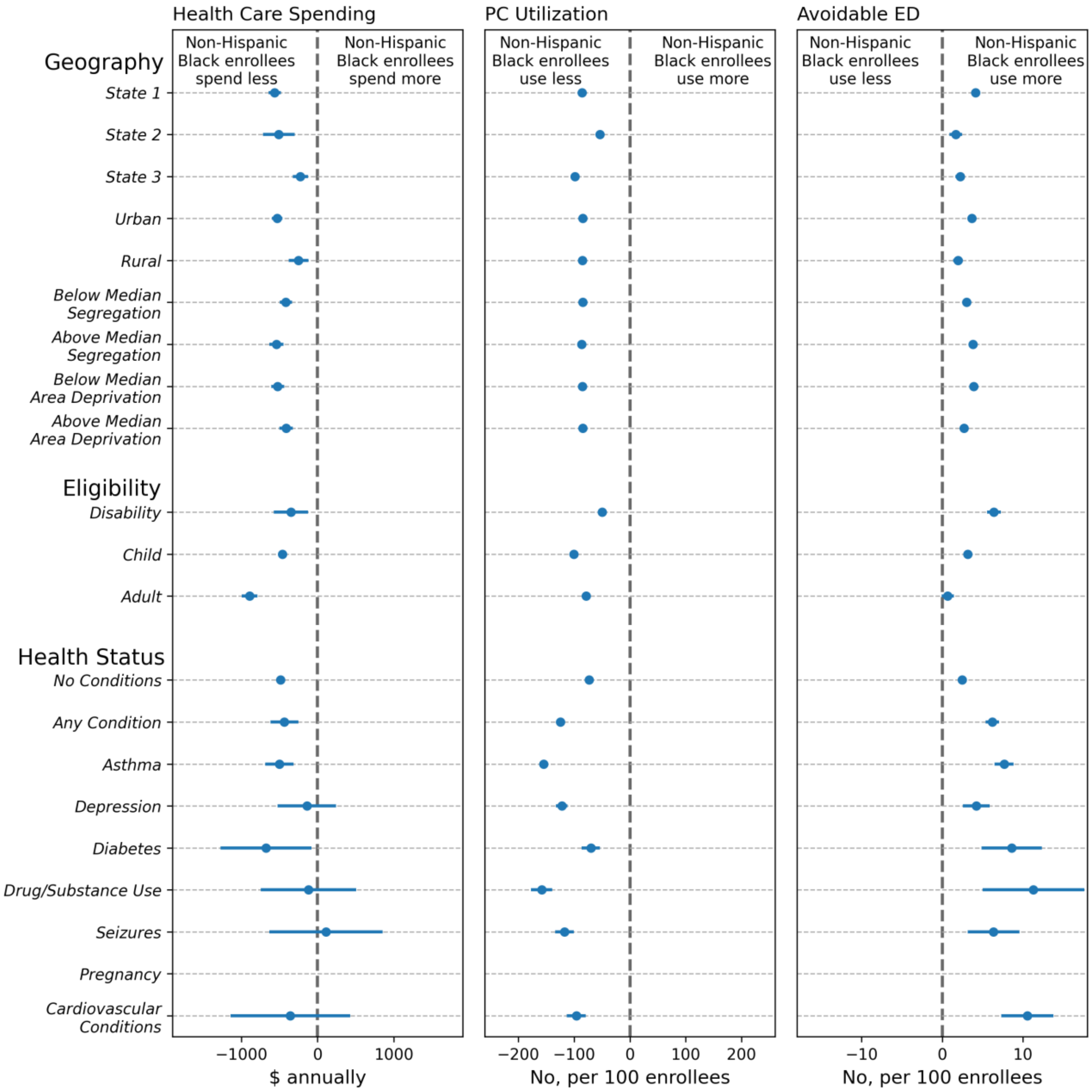

Legend: Estimates and 95% CIs for health care spending, primary care (PC) utilization, and avoidable emergency department (ED) visit rates for non-Hispanic Black men relative to non-Hispanic White men.

© 2022 Wallace J et al. *JAMA Health Forum.*

eTable 1. Sample state characteristics in 2016

Characteristics	Sample States, %				Rest of States, %	Difference [95% CI]	*P*-value [adjusted]
	State 1	State 2	State 3	Total			
Demographic Characteristics†							
% In poverty	20.2	12.3	16.0	16.1	13.4	2.7 [1.0, 4.5]	0.002 [0.007]
% College Educated	22.9	31.7	25.6	26.7	29.8	-3.0 [-7.0, 0.9]	0.13 [0.32]
# Active MD/DOs per 1,000 persons	3.5	3.2	3.5	3.4	3.6	-0.2 [-1.0, 0.5]	0.57 [0.88]
# Total Hospitals per 100,000 persons	5.1	6.3	2.6	4.7	3.1	1.6 [0.9, 2.2]	<0.001 [<0.001]
% Non-Hispanic Black	33.2	6.1	17.6	19.0	10.1	8.9 [3.4, 14.3]	0.001 [0.006]
% Non-Hispanic White	58.6	76.3	73.8	69.6	69.6	0.0 [-5.7, 5.6]	0.99 [0.99]
% Hispanic	4.5	11.7	5.2	7.1	11.9	-4.7 [-6.8, -2.7]	<0.001 [<0.001]
Geographic Designation†							
Metropolitan population	83.2	67.7	77.4	76.1	76.2	-0.1 [-8.1, 7.9]	0.98 [0.99]
Micropolitan population	9.6	19.2	13.0	13.9	13.7	0.3 [-5.5, 6.1]	0.92 [0.99]
Nonmetropolitan population	7.2	13.1	9.6	10.0	10.2	-0.2 [-3.8, 3.5]	0.92 [0.99]
*Medicaid coverage**							
Medicaid	25.3	13.9	21.3	20.2	19.8	0.4 [-5.4, 6.1]	0.9 [0.99]
Medicaid in managed care	84.0	89.7	92.4	88.7	67.7	21.0 [11.8, 30.2]	<0.001 [<0.001]
*Other health insurance overage**							
Employer coverage	43.3	53.6	46.6	47.8	49.9	-2.0 [-7.3, 3.3]	0.45 [0.78]
Non-group coverage	7.1	8.3	6.8	7.4	6.9	0.5 [-0.4, 1.4]	0.23 [0.49]
Medicare	12.7	13.4	14.3	13.5	13.8	-0.4 [-1.3, 0.6]	0.46 [0.78]
Military	1.5	2.3	1.9	1.9	1.8	0.1 [-0.4, 0.7]	0.62 [0.88]

© 2022 Wallace J et al. *JAMA Health Forum.*

Uninsured	10.2	8.4	9.1	9.2	7.9	1.3 [0.1, 2.6]	0.04 [0.12]

† Data are available at the county level – averages are calculated as county-level averages weighted by the share of each state's population contained within that county.
* Data are available at the state level – averages weight each state equally.

© 2022 Wallace J et al. *JAMA Health Forum.*

eTable 2. Self-reported racial and ethnic descriptions by state

Exact state specific self-reported race and ethnicity description	Share of state Medicaid population (pp)	Collective race and ethnicity term used in analysis
**State 1**		
American Indian or Alaskan Native	0.5	Native American or Alaska Native
Asian	1.3	Asian
Black or African American	44.8	Black
Hispanic or Latino and one or more other races	1.6	Hispanic
Hispanic or Latino (no other race info)	2.0	Hispanic
More than one race indicated (not Hispanic or Latino)	1.1	Other
Native Hawaiian or other Pacific Islander	0.2	Native Hawaiian or Pacific Islander
Not Declared	0.2	Other
Unknown	7.2	Other
White	41.1	White
**State 2**		
American Indian or Alaskan Native	1.6	Native American or Alaska Native
Asian	0.8	Asian
Black or African	12.8	Black
Pacific Islander including Native Hawaiian	0.1	Native Hawaiian or Pacific Islander
Unknown	15.5	Other
White	69.2	White
**State 3**		
American Indian or Alaskan Native	0.1	Native American or Alaska Native
Asian or Pacific Islander	0.8	Asian
Black	18.6	Black
Black (Non-Hispanic)	<0.1	Black
Caucasian	<0.1	White
Hispanic	3.7	Hispanic
Native Hawaiian	<0.1	Native Hawaiian or Pacific Islander
Not Provided	24.6	Other
Not Applicable	<0.1	Other
Other	7.9	Other
Pacific Islander	<0.1	Native Hawaiian or Pacific Islander
Subcontinent Asian American	0.1	Asian
White (Non-Hispanic)	44.1	White

© 2022 Wallace J et al. *JAMA Health Forum.*

Note: The exact state-specific self-reported term "other" used in State 3 was not further defined for us in the source data so we are unable to provide additional specificity.

© 2022 Wallace J et al. *JAMA Health Forum.*

eTable 3. Description of HEDIS Performance Measures

HEDIS Performance Measure	Description	Eligible Population
Breast Cancer Screening	Percentage of women ages 50 to 64 who had a mammogram to screen for breast cancer.	Women 50-64 years old.<br>Excludes women who had a bilateral mastectomy at any point in their history.
Cervical Cancer Screening	Percentage of women ages 24 to 64 who were screened for cervical cancer.	Women 24 -64 years old.<br>Excludes women who have a history of hysterectomy with no residual cervix, cervical agenesis, or acquired absence of cervix.
Chlamydia Screening in Women	Percentage of women ages 16 to 24 who were identified as sexually active and who had at least one test for chlamydia.	Women 16 to 24 years old who are identified as sexually active during the year.
Comprehensive Diabetes Care: HbA1c Testing	Percentage of enrollees ages 18 to 64 with diabetes (type 1 and type 2) who had a hemoglobin A1c (HbA1c) test.	Enrollees 18 to 64 years old who are identified as diabetic.
Diabetes Screening for people with Schizophrenia	Percentage of beneficiaries ages 18 to 64 with schizophrenia or bipolar disorder who were dispensed an antipsychotic medication and had a diabetes screening test during the measurement year.	Enrollees 18-64 years old identified as having schizophrenia or bipolar disorder AND diabetes
Asthma Medication Ratio	The percentage of enrollees ages 5 to 64 who were identified as having persistent asthma and had a ratio of controller medications to total asthma medications of 0.50 or greater.	Enrollees 5-64 years old identified as having persistent asthma.

© 2022 Wallace J et al. *JAMA Health Forum.*

eTable 4. Study population by state

	State 1		State 2		State 3	
	**White Enrollees (N=402,998)**	**Black Enrollees (N=585,284)**	**White Enrollees (N=241,998)**	**Black Enrollees (N=45,024)**	**White Enrollees (N=454,510)**	**Black Enrollees (N=236,875)**
**Demographic characteristics**						
Age, years (SD)	20.9 (17.4)	20.4 (17.0)	17.0 (15.8)	18.4 (16.6)	22.1 (17.7)	19.5 (16.7)
Female, No. (%)	228,183 (56.6)	337,474 (57.7)	129,319 (53.4)	24,231 (53.8)	260,464 (57.3)	139,465 (58.9)
Enrolled in MMC, No. (%)	359,565 (89.2)	525,341 (89.8)	236,091 (97.6)	43,849 (97.4)	454,510 (100.0)	236,875 (100.0)
**Geographic Characteristics**						
Urban, No. (%)	309,707 (76.9)	489,269 (83.6)	141,966 (58.7)	39,105 (86.9)	285,504 (62.8)	216,655 (91.5)
Residential Segregation, value (SD)	47 (11.7)	52 (11.9)	46 (11.7)	46 (9.3)	47 (13.6)	55 (12.1)
Area Deprivation Index, value (SD)	66 (16.1)	69 (15.9)	71 (17.0)	72 (18.5)	68 (14.1)	75 (17.4)
**Medicaid eligibility category, No. (%)**						
Disability	47,517 (11.8)	83,977 (14.3)	46,064 (19.0)	10,564 (23.5)	70,418 (15.5)	32,456 (13.7)
Child	231,102 (57.3)	326,965 (55.9)	165,615 (68.4)	28,369 (63.0)	244,758 (53.9)	144,404 (61.0)
Adult	97,728 (24.3)	135,866 (23.2)	25,663 (10.6)	5,270 (11.7)	107,084 (23.6)	51,306 (21.7)
Other	26,651 (6.6)	38,476 (6.6)	4,656 (1.9)	821 (1.8)	32,250 (7.1)	8,709 (3.7)
**Health Conditions, No. (%)**[†]						
Number of conditions, value (SD)	0.42 (0.97)	0.39 (0.92)	0.50 (1.10)	0.60 (1.22)	0.52 (1.11)	0.44 (1.00)
Number of conditions conditional,	1.67 (2.55)	1.58 (2.51)	1.78 (2.71)	1.77 (2.61)	1.81 (2.59)	1.68 (2.59)
Any Condition	102,366 (25.4)	145,381 (24.8)	67,661 (28.0)	15,242 (33.9)	130,908 (28.8)	62,185 (26.3)
Asthma/COPD	35,062 (8.7)	57,499 (9.8)	24,135 (10.0)	7,529 (16.7)	49,898 (11.0)	26,659 (11.3)
Cardiovascular Conditions	11,436 (2.8)	14,593 (2.5)	7,607 (3.1)	1,786 (4.0)	14,100 (3.1)	6,379 (2.7)
Depressive/Bipolar/Psychotic Disorders	26,588 (6.6)	25,459 (4.3)	21,620 (8.9)	3,709 (8.2)	31,074 (6.8)	11,144 (4.7)
Diabetes	12,103 (3.0)	22,442 (3.8)	9,552 (3.9)	2,352 (5.2)	19,656 (4.3)	10,310 (4.4)
Drug/Substance Use Disorders	11,319 (2.8)	7,633 (1.3)	5,743 (2.4)	1,189 (2.6)	18,313 (4.0)	4,252 (1.8)
Pregnancy	14,733 (3.7)	24,049 (4.1)	7,567 (3.1)	1,608 (3.6)	19,387 (4.3)	10,372 (4.4)
Seizures	9,390 (2.3)	10,365 (1.8)	7,027 (2.9)	1,056 (2.3)	11,599 (2.6)	3,999 (1.7)

[†]Based on HHS-HCCs. For purposes of reporting, individual HHS-HCCs are grouped into similar descriptive categories: Asthma/COPD (HCC 160, 161.1, 161.2), Cardiovascular Conditions (HCC 125, 126, 127, 128, 129, 130 131, 132, 135, 137, 138, 139, 142), Depressive/Bipolar/Psychotic Disorders (HCC 87.1, 87.2, 88, 90, 102, 103),

© 2022 Wallace J et al. *JAMA Health Forum.*

Diabetes (HCC 20, 21) Drug/Substance Use Disorders (HCC 81, 82, 83, 84), Pregnancy (HCC 203, 204, 205, 207, 208, 209, 210, 211, 212), Seizures (HCC 120).

© 2022 Wallace J et al. *JAMA Health Forum.*

eTable 5. Additional prescription drug quantity measures, including any fills within a year and medication possession ratio

	Unadjusted Means		Adjusted for Demographics	Adjusted for Demographics and Health Status	Adjusted for Demographics, Health Status, and Provider
	Black	White	Black-White Gap (95% CI)	Black-White Gap (95% CI)	Black-White Gap (95% CI)
Panel A: Adults	**(*N*= 344,023)**	**(*N*= 436,420)**	**(*N*= 780,443)**		
**Select Rx drug utilization, any utilization in a year conditional on having the condition**					
Anti-hypertensives†	74.81 (*N*=86,666)	69.3 (*N*=91,591)	4.49 [3.94, 5.04]	4.33 [3.77, 4.88]	4.58 [4.01, 5.15]
Asthma medication†	64.84 (*N*=31,224)	63.9 (*N*=56,440)	-2.13 [-3.01, -1.26]	-2.63 [-3.52, -1.73]	-2.32 [-3.26, -1.38]
Diabetes medication†	65.43 (*N*=33,303)	62.3 (*N*=39,129)	-0.18 [-1.12, 0.76]	-0.09 [-1.02, 0.84]	-0.58 [-1.56, 0.40]
Statins†	38.18 (*N*=16,500)	33.6 (*N*=23,881)	0.80 [-0.41, 2.00]	-1.84 [-3.00, -0.67]	-2.13 [-3.41, -0.86]
**Medication Possession Ratios, conditional on having the condition**					
Anti-hypertensives†	107.2 (*N*=64,833)	106.1 (*N*=63,473)	5.90 [4.85, 6.96]	3.74 [2.68, 4.80]	4.03 [2.91, 5.14]
Asthma medication†	62.5 (*N*=20,247)	68.6 (*N*=36,085)	-5.07 [-6.49, -3.65]	-5.32 [-6.76, -3.88]	-5.82 [-7.37, -4.28]
Diabetes medication†	96.4 (*N*=21,789)	114.8 (*N*=24,376)	-14.65 [-16.46, -12.84]	-15.14 [-16.95, -13.33]	-15.39 [-17.35, -13.44]
Statins†	65.7 (*N*=6,299)	72.0 (*N*=8,032)	-6.71 [-8.09, -5.32]	-7.40 [-8.82, -5.98]	-7.45 [-9.10, -5.81]
Panel B: Kids	**(*N*= 523,160)**	**(*N*= 663,086)**	**(*N*= 1,186,246)**		
**Select Rx drug utilization, any utilization in a year conditional on having the condition**					
Asthma medication†	84.22 (*N*=60,463)	85.0 (*N*=52,655)	-0.18 [-0.74, 0.38]	-0.31 [-0.87, 0.25]	0.37 [-0.21, 0.95]
Diabetes medication†	60.30 (*N*=1,801)	74.7 (*N*=2,182)	-7.90 [-12.18, -3.61]	-2.95 [-6.83, 0.93]	-1.70 [-6.23, 2.83]
**Medication Possession Ratios, conditional on having the condition**					
Asthma medication†	53.4 (*N*=50,920)	60.8 (*N*=44,765)	-1.95 [-2.86, -1.04]	-1.72 [-2.62, -0.81]	-1.54 [-2.48, -0.59]
Diabetes medication†	90.0 (*N*=1,086)	101.2 (*N*=1,630)	-2.08 [-9.08, 4.93]	3.99 [-2.83, 10.81]	1.71 [-8.62, 12.03]

© 2022 Wallace J et al. *JAMA Health Forum.*

[†]Drug groupings are defined using different levels of the Anatomical Therapeutic Chemical Classification (ATC) system. Anti-hypertensives are defined by ATC level 2 C02, C03, C07, C08 and C09 and exclude ATC C02KX01, C03BA08, C03CA01, C07AA07 and C07AA12; asthma medications are defined by ATC level 2 R03; diabetes medications are defined by ATC level 2 A10; and statins are defined by ATC level 4 C10AA. Measures are assessed for enrollees with related diagnosed conditions (see **eMethods 1** in the **Supplement**). For measures based on a subset of the population, sample sizes are presented under unadjusted means.

© 2022 Wallace J et al. *JAMA Health Forum.*

eTable 6. Robustness of racial disparities to transformations of health care spending

	Non-Hispanic White Enrollee Mean	Adjusted for Demographics	Adjusted for Demographics and Health Status	Adjusted for Demographics, Health Status, and Provider
		Black-White Gap (95% CI)	Black-White Gap (95% CI)	Black-White Gap (95% CI)
**Transformations of Total Health Care Spending**				
Log transformed spending (pp)	$3098*	-0.18 [-0.19, -0.17]	-0.13 [-0.14, -0.12]	-0.16 [-0.16, -0.15]
Spending winsorised at $40,000	$2692	-$424 [-$445, -$402]	-$286 [-$303, -$269]	-$265 [-$279, -$251]
Spending winsorised at $125,000	$3016	-$526 [-$559, -$492]	-$349 [-$377, -$321]	-$281 [-$303, -$258]

* For log transformed spending we report the mean for non-Hispanic White enrollees

© 2022 Wallace J et al. *JAMA Health Forum.*

REFERENCES

1. Hochberg Y. A sharper Bonferroni procedure for multiple tests of significance. *Biometrika*. 1988;75(4):800-802.
2. Benjamini Y, Hochberg Y. Controlling the false discovery rate: a practical and powerful approach to multiple testing. *J R Stat Soc Ser B.* 1995;57(1):289-300.

© 2022 Wallace J et al. *JAMA Health Forum.*
